# Bone morphogenetic protein 2 gene transduction enhances the osteogenic potential of human urine-derived stem cells

**DOI:** 10.1186/scrt539

**Published:** 2015-01-07

**Authors:** Junjie Guan, Jieyuan Zhang, Zhenzhong Zhu, Xin Niu, Shangchun Guo, Yang Wang, Changqing Zhang

**Affiliations:** Department of Orthopedics, Shanghai Jiao Tong University Affiliated Sixth People’s Hospital, 600 Yishan Road, Shanghai, 200233 China; Institute of Microsurgery on Extremities, Shanghai Jiao Tong University Affiliated Sixth People’s Hospital, 600 Yishan Road, Shanghai, 200233 China

## Abstract

**Introduction:**

Urine-derived stem cells (USCs) have the ability to differentiate into osteogenic lineage. Previous studies have raised the possibility that USCs could be used for bone repair. To harness the power of USCs in promoting bone regeneration, methods must be developed to induce USCs to osteogenic lineage efficiently. The present study investigates the effect of lentivirus-encoded bone morphogenetic protein 2 (BMP2) gene transduction on the osteogenic potential of USCs.

**Methods:**

USCs were isolated from voided urine and transduced with Lentiviral vector encoding BMP2. An *in vitro* study was performed to detect Lentiviral-BMP2 transduced USCs differentiated towards osteogenic lineage. Furthermore, Lentiviral-BMP2 transduced USCs were transplanted *in vivo* to examine the ectopic bone formation ability. After six weeks, retrieval samples were obtained for immunostaining and histological analysis.

**Results:**

The results showed that the transduction efficiencies were over 90%, and transduced USCs had high expression levels of the BMP2 gene and secreted BMP2 protein. Alkaline activity and mineral deposition staining demonstrated that transduced USCs differentiate into osteogenic lineages without the addition of osteogenic supplements. Transduced USCs also showed high expression of bone-related markers, including runt-related protein-2 (Runx2) and osteocalcin (OCN), confirming this lentiviral-BMP2 construct provides sufficient stimuli for osteogenic differentiation. Histological analysis indicated that the transduced USCs induced robust new bone formation in nude mice. Six weeks after transplantation, human derived cells were observed to participate in bone formation.

**Conclusions:**

These results demonstrate that BMP2 gene transduction provides an effective method to enhance the osteogenic potential of USCs.

## Introduction

Urine has recently been determined to be a source of adult stem cells. Zhang *et al*. reported that urine-derived stem cells (USCs) can be expanded *in vitro* and differentiated into multiple cell lineages [1]. Our previous studies demonstrated that USCs express similar surface markers as adipose-derived stem cells (ASCs). Under certain induction conditions, USCs can differentiate into osteoblasts, chondrocytes and adipocytes [2]. Compared with other cell sources, urine is vastly available and can be obtained by non-invasive methods. The potential for USCs-based cytotherapy has gained the attention of researchers. USCs seeded onto bacterial cellulose could express urothelial markers [3]. USCs with a small intestinal submucosal scaffold could form tissue that is similar to the native urethral structure [4]. Those results demonstrated that USCs hold promise for use in urinary construction. We first combined USCs and a β-tricalcium phosphate (β-TCP) scaffold to heal critically sized bone defects in rats (data unpublished). The results showed that USCs hold tremendous promise for bone tissue engineering; however, there is considerable room to enhance their repair ability. An alternative approach to promote the osteogenic potential of USCs may be to transduce them so that they express growth factors that direct USCs differentiation toward the osteoblasts cell fate. Bone morphogenetic protein 2 (BMP2), an essential growth factor for bone formation, is a possible candidate for this application.

BMP2 belongs to the transforming growth factor beta superfamily and has been used in spinal fusions, long bone defects and non-union bone fractures [5]. However, human trials have been less successful than animal studies. Researchers have found that the low biological activity and short *in vivo* half-life of BMP2 may cause this reduced success [6]. In addition, recombinant BMP2 protein is quite expensive and would represent an increased health cost for treatment [7]. Therefore, researchers have turned to gene therapy as an approach to deliver therapeutic proteins in a more persistent and physiological manner. ASCs can be transduced with the BMP2 gene to form bone *in vivo*[8]. Wright *et al*. transduced a retrovirus encoding BMP2 into muscle-derived stem cells (MDSCs), and their results demonstrated that MDSCs undergo osteogenic differentiation. Furthermore, the transduced MDSCs induce significant new bone formation [9]. Currently, it is still unknown whether BMP2 gene transduction can improve the osteogenic ability of USCs.

The aims of present study were to: (1) determine if human USCs can be efficiently transduced with a lentivirus encoding BMP2; (2) determine if these transfected cells can undergo osteogenic differentiation; and (3) determine if transduced USCs can be used to form bone in vivo.

## Methods

### Isolation of human USCs

Primary USCs were harvested from five healthy adult donors (male, age range 23 to 30 years) using methods described previously [2]. The Ethics Committee of Shanghai Six People’s Hospital approved the use of human urine. All volunteers signed the informed consent. A total of 200 ml sterile urine samples were centrifuged and washed with 80 ml phosphate-buffered saline (PBS), and cell pellets were resuspended and plated in 24-well plates with mixed culture medium. The medium included (Dulbecco’s) modified Eagle’s medium ((D)MEM) culture medium supplemented with 2% (vol/vol) fetal bovine serum (FBS) (Gibco, Invitrogen, Grand Island, NY, USA), 10 ng/ml human epidermal growth factor (hEGF, Peprotech, NJ, USA), 2 ng/ml platelet-derived growth factor (PDGF, Millipore, MA, USA), 1 ng/ml transforming growth factor-β (TGF-β, Peprotech), 2 ng/ml basic fibroblast growth factor (bFGF, Sigma-Aldrich, St. Louis, MO, USA), 0.5 μM cortisol (Sigma-Aldrich), 25 μg/ml insulin (Humulin, Eli Lilly, Indianapolis, USA), 20 μg/ml transferrin, 549 ng/ml adrenaline, 50 ng/ml triiodothyronine, L-glutamine and antibiotics. Five to seven days later, the non-adherent cells were washed out using PBS. Medium was changed every three days. After reaching subconfluence, the cells were passaged using trypsin.

### Characterization of USCs

#### Surface marker expression of the USCs

To detect the surface antigens expressed on USCs, the cells were subjected to flow cytometry analysis. Briefly, USCs were harvested, washed and incubated with the following fluorochrome-conjugated antibodies: CD29, CD34, CD44, CD45, CD73, CD90, CD133 and HLA-DR for 30 minutes at 4°C (all from BD, Biosciences, CA, USA). The negative control received an equivalent amount of isotype-matched antibodies. Flow cytometry was performed using a Guava Technologies flow cytometer (Guava, Easy Cyte HT,MA, USA). The data were analyzed using Cytosofe (Version 5.2, Guava Technologies).

#### Multilineage differentiation potential

USCs were seeded onto six-well plates. The osteogenic medium (Invitrogen, Gibco) was added when cells reached 90% confluence and the medium was replaced twice a week. After 21 days of osteogenic differentiation, calcium deposition was detected by Alizarin Red S staining. To induce adipogenic differentiation, adipogenic medium was added when cells reached 90% confluence (Invitrogen, Gibco), and the medium was replaced twice a week. Intracellular lipid droplets were confirmed by Oil Red O staining.

#### Construction of the lentiviral vector

Lentiviral vectors carrying human BMP2 and green fluorescent protein (GFP) were constructed. One vector encoded BMP2 and GFP. However, the second vector only encoded GFP. Total RNA was harvested using the PrimeScript RT reagent kit according to the manufacturer’s instructions (TAKARA-BIO, Shiga, Japan). The RNA was converted into complementary DNA (cDNA) using a Reverse Transcription System Kit (Invitrogen, Carlsbad, CA, USA). Based on the cDNA sequences of BMP2 from the Genebank database (Gen-Bank, Accession No.NM001200), the primers were synthesized using Primer Premier 5.0. software. The BMP2 gene was amplified by PCR and inserted into the LV5 vector (GenePharma Co., Ltd, Shanghai, China). The Cytomegalovirus (CMV) promoter was used to drive gene expression. The shuttle vector and packaging plasmids pGag/Pol, pRev and pVSV-G were transfected into 293 T cells for lentiviral production. The viruses were collected on Day 3 after the transfection and were concentrated by ultracentrifugation.

#### Gene transduction

USCs were trypsinized, centrifuged and counted when they reached 70% confluence. They were exposed to lentiviruses at a multiplicity of infection (MOI) of 10, 50 and 100. A total of 8 μg/ml Polybrene (Sigma) was added to enhance the transduction efficiency. The transduction medium was replaced with basal medium after 48 hours.

#### Gene transduction efficiency and cell viability assay

Cells were observed under a fluorescence microscope three days after transduction (Nikon, ECLIPSE, Ti). The gene transduction efficiency was further analyzed using flow cytometry (Guava, easy Cyte HT). The cells were trypsinized and centrifuged before analysis. Then cells were subjected to flow cytometry (Guava easyCyte™). The percentage of GFP-positive cells was analyzed using Cytosofe (Version 5.2, Guava Technologies).

Cell viability was evaluated using a CCK-8 assay kit according to the manufacturer’s instructions. In brief, 5,000 cells were plated on a 96-well assay plate containing 100 μL culture medium. At 1, 3 and 7 days after plating, 10 μL CCK-8 solution was added, and the plate was incubated for two hours at 37°C in a humidified, 5% carbon dioxide atmosphere. The absorbance at 450 nm was then recorded using a microplate reader (iMark^™^; Bio-Rad, Hercules, CA, USA).

#### Quantitative RT-PCR analysis

Quantitative RT-PCR was performed to detect the related genes expression in Lentiviral-BMP2 transduced USCs at 3, 7 and 14 days after transduction. Total RNA was isolated from the cells using the Trizol method (Invitrogen). After the reverse transcription reaction, quantitative real-time PCR was carried out using TaqMan Universal PCR Master Mix (Roche, Branchburg, NJ, USA). The product was quantified using a standard curve, and GAPDH was used as control. The sequences for the primers used are listed in Table 1. Normal USCs and Lentiviral-GFP transduced USCs were analyzed as control. Each assay was performed in triplicate.

**Table 1:** **Primers used in RT-PCR and production sizes**

		Amplication	Ann.temp
Gene	Primer sequence (5′-3′)	Size	(°C)
BMP2	Forward GAGAAGGAGGAGGCAAAGAAA	161	56
	Reverse AGCAGCAACGCTAGAAGACAG		
OCN	Forward CCCCCTCTAGCCTAGGACC	151	56.4
	Reverse ACCAGGTAATGCCAGTTTGC		
Runx2	Forward CCAACCCACGAATGCACTATC	71	58.9
	Reverse TAGTGAGTGGTGGCGGACATAC		
GAPDH	Forward ATCCCATCACCATCTTCC	293	51
	Reverse GAGTCCTTCCACGATACCA		

#### BMP2 ELISA

The secretion of BMP2 was measured in the culture medium of normal USCs, Lentiviral-GFP transduced USCs and Lentiviral-BMP2 transduced USCs. Briefly, the cells were trypsinized and seeded at a density 1 × 10^5^ cells/well in six-well plates. Culture medium was collected and underwent analysis according to manufacturer’s instruction (Quantikine, R&D Systems, Minneapolis, MN, USA). The protein content was determined with a BCA protein assay kit (Pierce Biotechnolog, Rockford, IL, USA). All experiments were performed in triplicate.

#### Osteogenic differentiation of Lentiviral-BMP2 transduced USCs

To investigate the effect of inductive osteogenesis by gene transfection only, lentiviral-BMP2 transduced USCs were cultured in basal medium as described in the Section 'Isolation of human USCs’. The medium was replaced every three to four days. Osteogenic differentiation was assessed by measuring alkaline phosphatase (ALP) activity, mineral calcium deposition, and gene and protein expression of bone-relate markers.

#### Alkaline Phosphatase activity

On days 7 and 14, cells were washed twice with PBS and then 0.1% Triton X-100 was added to dissolve the cells. The solution was transferred into a 1.5 mL tube, and the samples were then centrifuged at 14,000 rpm at 4°C for 20 minutes. The supernatants were transferred to fresh 1.5 mL tubes and ALP reaction buffer (100 μl 1 M Tris–HCl, 20 μl 5 mM MgCl_2_, and 20 μl 5 mM p-nitrophenyl phosphate) was added. After incubation for 30 minutes at 37°C the reaction was stopped by the addition of 50 μL of 1 N NaOH. Using p-nitrophenol as a standard, the absorbance was measured at 410 nm with a spectrophotometer. The ALP activity was expressed as the value of the p-nitrophenol production quantity divided by the reaction time and the protein synthesis quantity, as measured by a BCA Protein assay kit (Thermo Scientific, Rockford, IL, USA).

#### Mineral calcium deposition

After 14 days of incubation, calcium deposition was detected by Alizarin red S and von Kossa staining. Briefly, cells were washed twice with PBS and fixed with 4% paraformaldehyde (PFA) for 20 minutes. The fixed cells were then washed with PBS. For the Alizarin red S staining, the cells were treated with 2% Alizarin Red S for 15 minutes at room temperature. After the removal of unincorporated dye with distilled water, the cells were observed under a microscope. For the von Kossa staining, cells were fixed in cold methanol for 20 minutes. After washing with PBS, the cells were incubated with 5% silver nitrate solution under UV light for 30 minutes. The cells were washed with PBS and treated with a 5% solution of sodium thiosulfate (Sigma-Aldrich, St. Louis, MO, USA). The mineralized nodules were labeled as black spots.

#### Gene and protein expression of osteogenic markers OCN and Runx2

The effect of BMP2 gene transduction on the gene expression of bone-related markers Runx2 and OCN was measured by RT-PCR. RT-PCR was performed as described before in 'Quantitative RT-PCR analysis’.

After 7 and 21 days of culture, cells were washed in PBS and fixed with 4% PFA in PBS at room temperature for 15 minutes. After that, the samples were rinsed in PBS and permeabilized in 0.1% Triton X-100 for 10 minutes. Non-specific antigen binding was blocked with 3% BSA/PBS at 37°C for 20 minutes. Subsequently, the cells were incubated with primary antibody against Runx2 (Abcam, Cambridge, UK, ab76956, 1:200) and OCN (Abcam, Cambridge, UK, ab13420, 1:200) overnight at 4°C. After washing with PBS, the cells were incubated with Alexa Fluor 594 goat anti-mouse secondary antibody (Invitrogen, 1:200) for two hours at 37°C. Finally, the nuclei were stained with 4',6-diamidino-2-phenylindole (DAPI), and the image was analyzed with a fluorescence microscope.

#### USCs transplantation and in vivo bone formation

All procedures were approved by the Shanghai Sixth People’s Hospital Committee on the Use and Care of Animals. USCs and Lentirival-BMP2 transduced USCs were seeded onto a β-TCP scaffold. Two muscle pockets were made and two different implants were placed on each mouse. Twelve implants were placed and six mice were needed for the whole *in vivo* experiments. Transplantation surgery was performed as described previously [7]. Briefly, nude mice were adequately anesthetized with 10% chloral hydrate (0.35 to 0.4 mL/100 g). A skin incision was made on the right hindlimbs, and muscle pockets were formed by blunt dissection. The composite was implanted into the musculature, and the incisions were closed with 4–0 silk suture. Animals were allowed activity *ad libitum*.

Six weeks after implantation, the transplants were harvested from muscles, fixed in 10% formalin neutral buffer solution and decalcified in 10% ethylenediaminetetraacetic acid (EDTA). The specimens were then dehydrated through a series of graded ethanol, infiltrated and embedded in paraffin wax. The tissues were cut into 6 μm sections and stained with hematoxylin and eosin (H & E). The sections were observed and photographed with a microscope (TY9648; Leica).

For immunohistochemical staining, paraffin-embedded sections were deparaffinized, hydrated and incubated with 3% hydrogen peroxide, and then boiled in sodium citrate buffer for 10 minutes. After the sections were cooled at room temperature, the slides were blocked for 30 minutes with 1.5% horse serum. Subsequently, the slides were incubated with the primary BMP2 antibody (Abcam, ab6285, 1:200 dilution) or collagen I (Col I) (Sigma, C2456, 1:1000 dilution) overnight at 4°C. Next, the slides were incubated with biotinylated secondary antibody for 30 minutes at room temperature. Counterstaining was performed with hematoxylin. The slides were observed by three individuals who were blinded to the treatments.

To confirm the implantation of the transfected USCs in the β-TCP scaffold and their survival, sections were analyzed with a fluorescence microscope (Nikon, ECLIPSE, Ti). Briefly, the sections were treated with 0.2% Triton X-100 for 20 minutes. After washing, the sections were blocked with 5% normal goat serum in PBS for 30 minutes at room temperature. The sections were washed three times with PBS, and then incubated with the primary antibody OCN (Abcam, ab13420, 1:200 dilution) at 4°C overnight, followed by incubation with Alexa Fluor 594 goat anti-mouse secondary antibody (Invitrogen, 1:200) for two hours. The sections were then stained with DAPI, and the slides were observed under a fluorescence microscope (MZFL III; Leica).

### Statistical analysis

All quantitative data were expressed as means ± standard deviations. Statistical analysis was performed via one-way analysis of variance (ANOVA) using SPSS software (SPSS Inc., Chicago, IL, USA). A value of *P* <0.05 was considered statistically significant.

## Results

### USCs characterization

**Figure 1:**
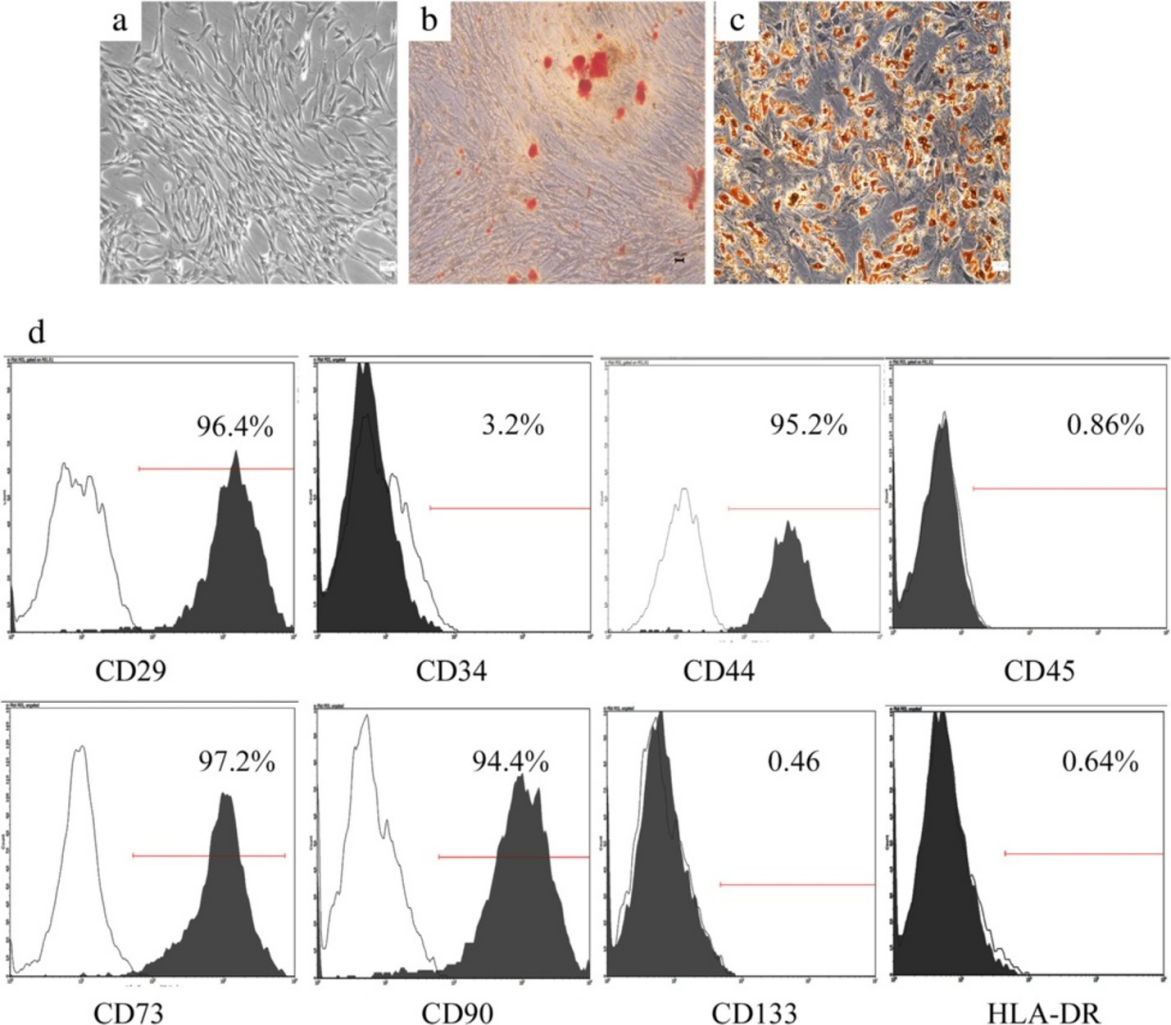
**Characterization of human urine-derived stem cells. (a)** Phase-contrast microscopy of USCs in primary culture. **(b)** Alizarin Red S staining showing the osteogenic differentiation of USCs. **(c)** Oil red O staining showing the adipogenic differentiation of USCs. **(d)**. Flow cytometry analysis of the expression of USCs surface markers. Scale bars = 100 μm. USCs, urine-derived stem cells.

USCs were isolated successfully from voided urine. The number of live cells in urine samples was 4.5 × 10^3^/100 ml urine (range 3.2 to 8.5 × 10^3^). Most living cells in these samples did not attach to culture plates and were discarded when the culture media was replaced. Generally, 100 ml urine contained 1 to 2 progenitor cells. They showed a fibroblastic morphology (Figure 1a), which is a similar finding as those of our previous results. USCs can be induced to differentiate into osteoblast and adipocyte lineages. Alizarin Red S staining confirmed the osteogenic differentiation (Figure 1b). Oil Red O staining showed the lipid vesicles in cytoplasm of USCs, which demonstrated the adipogenic differentiation (Figure 1c).

The immunophenotypes of the USCs are shown in Figure 1d. USCs positively expressed CD29, CD44, CD73 and CD90. However, they were negative for CD34, CD45, CD133 and HLA-DR.

### Transduction conditions and efficiency

**Figure 2:**
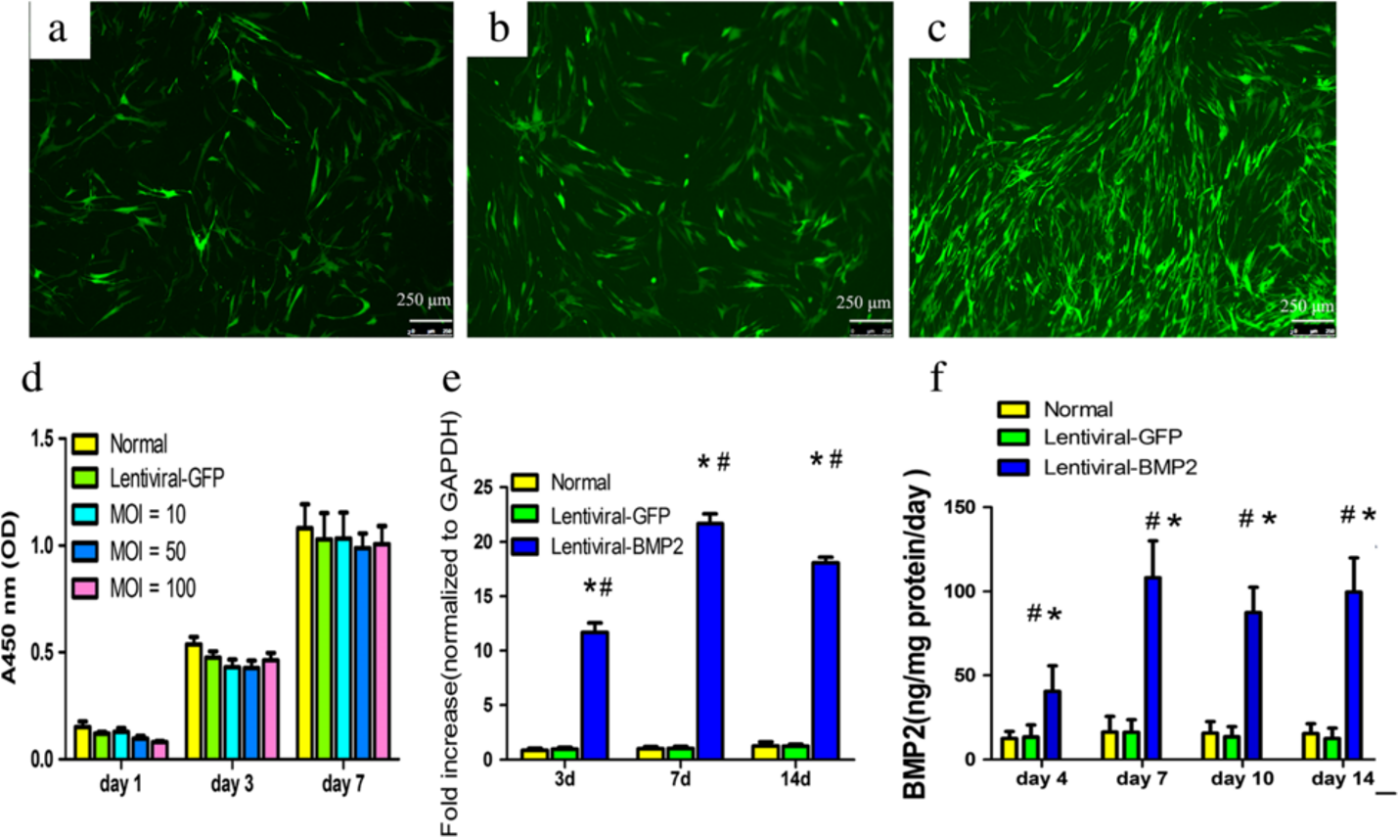
**Lentiviral-BMP2 transduced USCs and the effect of transduction on USCs proliferation and BMP2 gene and protein expression.** USCs with Lentiviral-BMP2 vectors at different MOIs (**a**:10; **b**:50; **c**:100). Cells were assessed for the presence of positive GFP fluorescence microscopy three days after transduction. Scale bar = 250 μm. **(d)** Cell proliferation assays were performed at 1, 3 and 7 days post-transduction. The means and standard deviations were calculated from three experiments. **(e)** RT-PCR analysis showed that the transduced USCs highly expressed the BMP2 gene at days 3, 7 and 14 after transduction. **(f)** ELISA results indicated that BMP2 production in Lentiviral-BMP2 transduced USCs was significantly increased as compared to that in Lentiviral-GFP transduced USCs or normal USCs. #, P <0.01 (compared with normal USCs) and *, *P* <0.01 (compared with Lentiviral-GFP transduced USCs). BMP2, bone morphogenic proteins 2; MOI, multiplicity of infection; USCs, urine-derived stem cells.

The Lentiviral-BMP2 transduction conditions were optimized by considering both transduction efficiency and cell viability. As expected, the percentage of GFP-positive cells increased with the increased MOI (Figure 2a, b, and c). Transduction efficiency was further evaluated by flow cytometry three days after transduction. As shown in Table 2, the percentage of GFP-positive cells increased as the MOI increased.

To determine the viability of USCs following Lentiviral gene transduction, CCK-8 analysis was performed. Figure 2d shows that Lentiviral-GFP and Lentiviral-BMP2 transduction had no significant effect on cell viability. We concluded that the optimized Lentiviral-BMP2 transduction MOI was 100 because it resulted in the highest GFP-positive rate and had no significant effect on cell viability. All of our following experiments were performed at an MOI of 100.

**Table 2:** **The percentage of GFP-positive cells at different MOI**

MOI	Samples number	Positive rate
10	3	30 ± 6%
50	3	53 ± 4%
100	3	90 ± 7%

MOI, multiplicity of infection.

### Expression of BMP2 gene and protein

To examine the expression of BMP2 in Lentiviral-BMP2 transduced USCs, cells were harvested and analyzed by RT-PCR. Lentiviral-BMP2 transduction significantly impacted the amount of BMP2 gene expression (Figure 2e). The results demonstrated the stable expression of BMP2 in Lentiviral-BMP2 transduced USCs. The amount of BMP2 protein secreted by these transduced cells was quantified using an ELISA assay kit (Figure 2f). The mean secretion of BMP2 over 4, 7, 10 and 14 days was 40.5 ± 21.5, 108.1 ± 31.2, 87.4 ± 21.5 and 99.7 ± 28.7 ng/mg protein/day in Lentirival-BMP2 transduced USCs, respectively.

### *In vitro*osteogenic induction

#### ALP activity assay and mineral calcium staining

**Figure 3:**
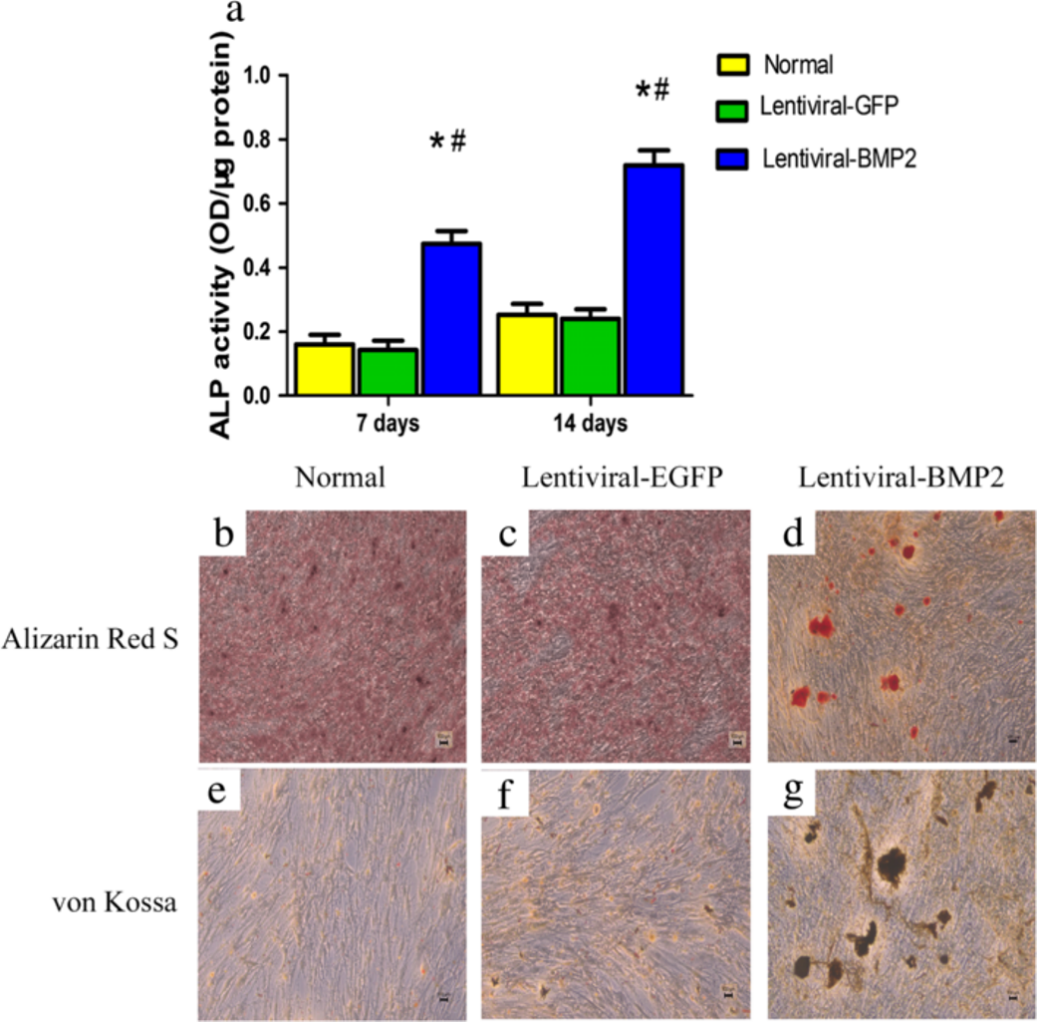
***In vitro*** **osteogenic differentiation of lentivirus-transduced USCs. (a)** ALP activity in Lentiviral-BMP2 transduced USCs was significantly higher than that in Lentiviral-GFP transduced USCs and normal USCs. The deposited calcium was detected using Alizarin Red S **(b, d and d)** and von Kossa staining **(e, f and g)** at 14 days after transduction. **(b and e)** Normal USCs. **(c and f)** Lentiviral-GFP transduced USCs. **(d and g)** Lentiviral-BMP2 transduced USCs. For Alizarin Red S staining, the red color indicates calcium deposition **(d)**; for von Kossa staining, dark patches indicate calcium deposition **(g)**. Scale bar = 100 μm. #, *P* <0.05 (compared with normal USCs) and *, *P* <0.05 (compared with Lentiviral-GFP transduced USCs). ALP, alkaline phosphatase; BMP2, bone morphogenic proteins 2; USCs, urine-derived stem cells.

We assessed osteogenic differentiation by measuring ALP activity and mineralization. As shown in Figure 3a, the ALP activity of the Lentiviral-BMP2 transduced USCs increased gradually over 14 days of cell culture and was significantly higher than that of the normal USCs and Lentiviral-GFP transduced USCs (*P* <0.05).

Fourteen days after transduction, Alizarin Red S and von Kossa staining showed that the mineralized nodules were significantly greater in Lentiviral-BMP2 transduced USCs than in normal USCs and Lentiviral-GFP transduced USCs (Figure 3b,c,d,e, f and g). Taken together, these results demonstrate that Lentiviral-BMP2 transduction enhances the osteogenic activity of USCs.

#### Expression of osteogenic related marker OCN and Runx2

**Figure 4:**
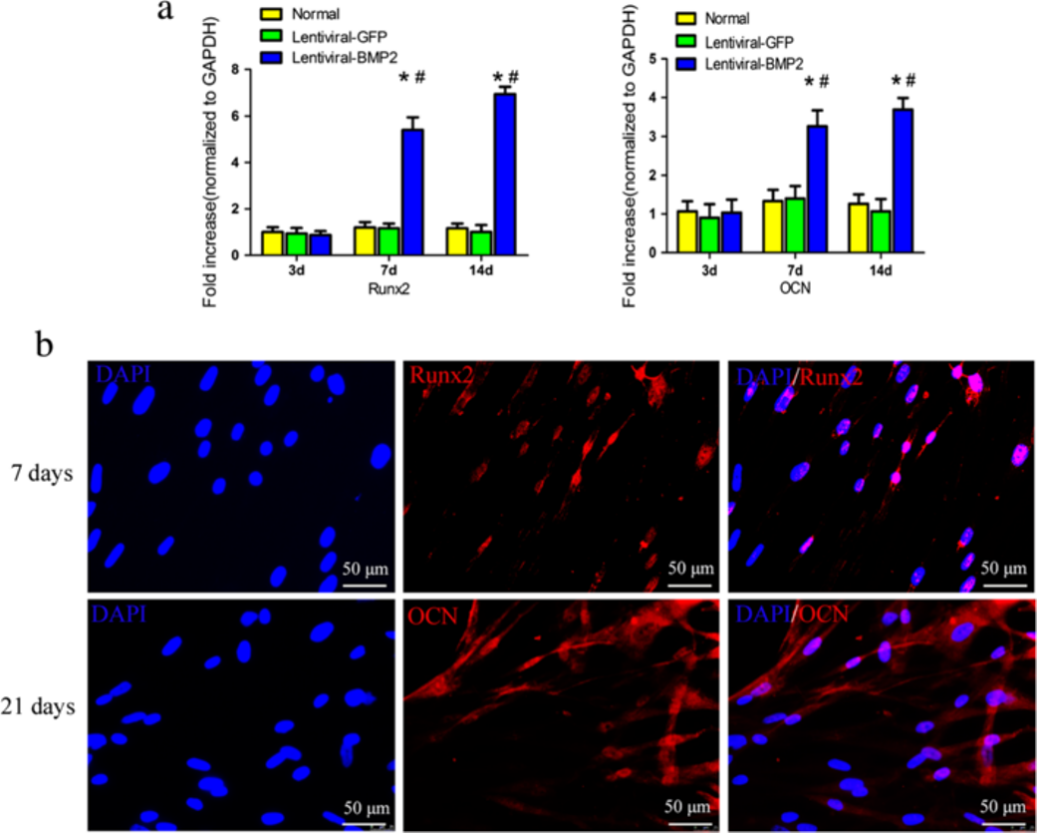
**Gene and protein expression of bone related factors in transduced and normal USCs. (a)** The mRNA expression level of Runx2 and OCN at day 3, 7 and 14 after transduction. **(b)** The protein expression of Runx2 and OCN was detected with immunofluorescence. Blue: DAPI; red: Runx2 or OCN. BMP2 gene transduction significantly increased the protein expression of Runx2 and OCN. Scale bar = 50 μm. #, *P* <0.05 (compared with normal USCs) and *, *P* <0.05 (compared with Lentiviral-GFP transduced USCs). BMP2, bone morphogenic proteins 2; DAPI, 4',6-diamidino-2-phenylindole; OCN, osteocalcin; Runx2, Runt-related protein-2; USCs, urine-derived stem cells.

Expression of Runx2 and OCN genes in Lentiviral-BMP2 transduced USCs was detected by RT-PCR on days 3, 7 and 14. Transcription level of these genes was significantly higher in Lentiviral-BMP2 transduced USCs than in normal USCs or Lentiviral-GFP transduced USCs (Figure 4a). To further detect the osteogenic differentiation of Lentiviral-BMP2 transduced USCs resulting from gene transduction, we used immunofluorescence to analyze the osteogenic-related proteins Runx2 and OCN. The results showed that Runx2 and OCN were expressed in Lentiviral-BMP2 transduced USCs at 7 and 21 days after transduction (Figure 4b), respectively. We conclude that the transduction of Lentiviral-BMP2 enhances the osteogenic differentiation of USCs *in vitro*.

#### In vivo bone formation

**Figure 5:**
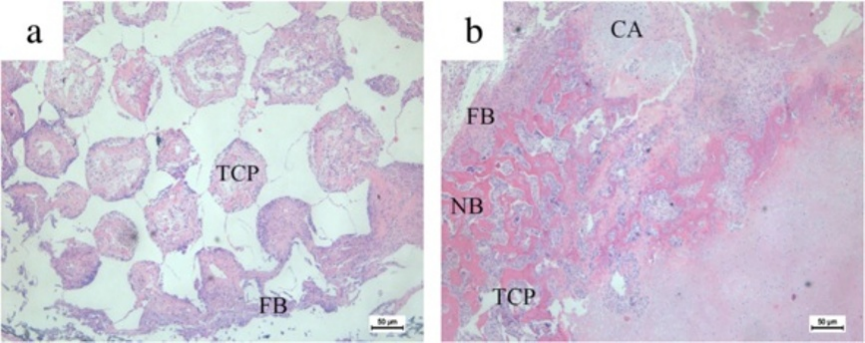
**H & E staining of histological sections formed by the implantation of Lentiviral-BMP2 transduced USCs/β-TCP and USCs/β-TCP into the hind limb of athymic mice for six weeks. (a)** The USCs/β-TCP induced no bone formation, whereas the Lentiviral-BMP2 transduced USCs/β-TCP resulted in bone formation in the outer surface and inner pore of the implants **(b)**. Scale bar = 50 μm. TCP: β-TCP; FB: fibroblast tissue; NB: new bone; CA: cartilage tissue. B-TCP, β-tricalcium phosphate; BMP2, bone morphogenic proteins 2; USCs, urine-derived stem cells.

To further test the osteogenic potential of Lentiviral-BMP2 transduced USCs *in vivo*, Lentiviral-BMP2 transduced USCs were seeded on β-TCP scaffolds and transplanted into the hind limbs of nude mice. Six weeks after *in vivo* implantation, mice were sacrificed and samples were collected and examined. H & E staining revealed that Lentiviral-BMP2 transduced USCs generated bone tissues, whereas little ectopic bone formation was observed in control mice implanted with normal USCs (Figure 5a and b). The results showed Lentiviral-BMP2 transduction dramatically increased USCs osteogenic activity relative to USCs control *in vivo*.

**Figure 6:**
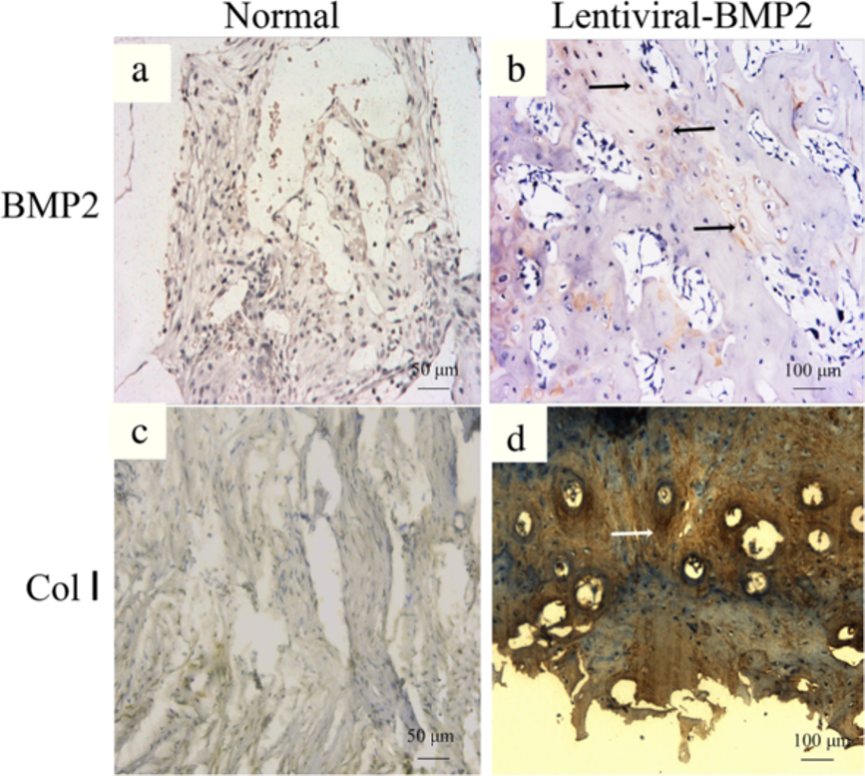
**Immunohistochemical staining of BMP2 and Collagen I of the transplanted scaffold at six weeks post-implantation. (a)** BMP2 and **(c)** Collagen I staining in the normal USCs transplantation group. **(b)** BMP2 and **(d)** Collagen I staining in the Lentiviral-BMP2 transplantation group. The arrows indicate the positive staining of BMP2 or Collagen I in the Lentiviral-BMP2 transplantation group. Scale bar for a and c = 50 μm; b and d = 100 μm. BMP2, bone morphogenic proteins 2; USCs, urine-derived stem cells.

Immunohistochemistry targeting BMP2 and Collagen I (Col I) was performed. It revealed the negative staining of BMP2 and Col I in the USCs transplantation group (Figure 6a and c). However, positive immunohistochemical staining for BMP2 and Col I was observed in the Lentiviral-BMP2 transduced USCs transplantation group (Figure 6b and d).

**Figure 7:**
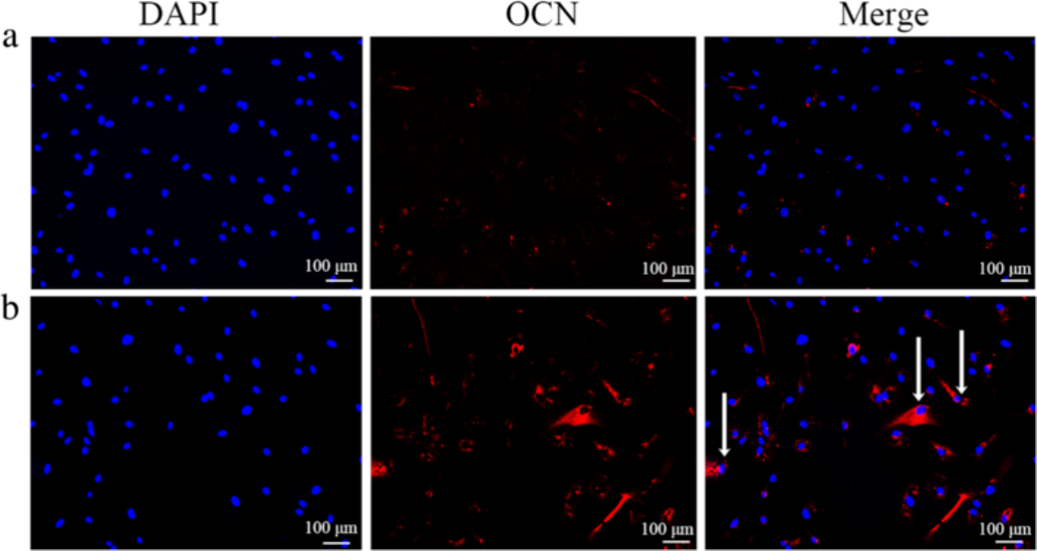
**Human derived OCN was detected by immunostaining after six weeks. (a)** USCs/β-TCP; **(b)** Lentiviral-BMP2 transduced USCs/β-TCP. The arrows indicate the positive staining of OCN. Scale bar = 100 μm. B-TCP, β-tricalcium phosphate; BMP2, bone morphogenic proteins 2; OCN, osteocalcin; USCs, urine-derived stem cells.

OCN immunofluorescence was performed to determine the osteogenic differentiation of USCs with or without Lentiviral-BMP2 transduction. Human derived OCN stained in red in the Lentiviral-BMP2 transduction group (Figure 7a). However, no human derived OCN was detected in the normal USCs transplantation group (Figure 7a). This result indicated that Lentiviral-BMP2 transduced USCs were positive for the osteoblast marker OCN, which suggested that Lentiviral-BMP2 transduced USCs differentiate into osteoblasts in response to the secreted BMP2.

## Discussion

In the present study, we demonstrated that the osteogenic activity of USCs can be enhanced by the gene transduction of Lentiviral-BMP2. After *ex vivo* transduction, Lentiviral-BMP2 transduced USCs can secrete high levels of BMP2 and induce *de novo* bone formation *in vivo*. To the best of our knowledge, this study is the first to evaluate the transduction of the BMP2 gene into USCs and the effect of gene transduction on their osteogenic ability.

USCs are adult stem cells that have been characterized in recent years [1]. USCs have the capacity to differentiate into osteoblasts, chondrocytes, adipocytes, muscle and neurons. The large source for the cells and non-invasive method required to collect them has made USCs a suitable choice for tissue engineering, including bone tissue engineering. The bone marrow is regarded as the natural reservoir of osteoprogenitor cells, and bone marrow-derived mesenchymal stem cells (BM-MSCs) are widely used to deliver growth factors for bone regeneration [10–12]. Nevertheless, bone marrow aspiration requires invasive surgery and causes additional pain to patients; these factors have impeded the widespread use of the bone marrow for these applications. Therefore, cell sources that are easily accessible and relatively abundant have a priority for cytotherapy. Due to their non-invasive procurement method and their high self-renewal capacity, USCs have gained increasing attention.

In the current study, we used culture media free of fetal bovine serum. Previous studies have demonstrated that various in the serum composition [13]. Additionally, the use of animal products has a high risk of virus transmission [14]. Currently, the serum-free media we used is mainly supplemented with approximately essential components including: insulin transferrin and selenium (ITS supplement), transferrin, glucocorticoids, triiodothyronine (T3), fibroblast growth factor-2 and glutamine. We combined these supplement depend on the healthy expansion of mesenchymal stem cells and the source environment of USCs [15]. The fetal bovine serum free media pave the way for clinical application of USCs.

Stem cell-based gene therapy is a promising tool to enhance bone repair. Substantial studies have demonstrated that BMP2 gene transduction can be used to enhance the bone formation ability of stem cells [7, 16, 17]. Our goal is to utilize BMP2 gene transduction to promote the osteogenic potential of USCs. We integrated the BMP2 gene into lentiviral vectors and transduced them into USCs with an MOI of 10, 50 and 100. The results showed that at an MOI of 100, over 90% of the USCs were transduced, which indicates a high transduction efficiency. In addition, the transduction process was found to have no significant effect on cell viability.

After *in vitro* transduction, USCs expressed high levels of the BMP2 gene and secreted a large amount of BMP2 protein. Furthermore, we present evidence that the secreted BMP2 promoted USCs differentiation into osteoblasts. ALP activity and mineral calcium deposition is a useful marker for osteogenic differentiation. Only osteoblasts or cells with osteoblastic characteristics produce OCN [18]. ALP activity and mineral calcium deposition increased significantly after transduction with Lentiviral-BMP2. Lentiviral-BMP2 transduction also stimulated the gene and protein expression of Runx2 and OCN. These data showed that the BMP2 production was sufficient to lead to robust osteogenesis by USCs. Previous studies have demonstrated that BMP2 secreted by transduced cells can initiate a physiological effect on stem cells [12]. As expected, the transduction of the BMP2 significantly enhanced the osteogenic differentiation of USCs *in vitro*.

We investigated whether Lentiviral-BMP2 transduced USCs combined with porous ceramic scaffolds made of β-TCP could induce ectopic bone formation *in vivo*. When scaffolds were seeded with Lentiviral-BMP2 transduced USCs, bone was observed six weeks after implantation. The control scaffold with USCs failed to form bone, indicating the crucial role of the Lentiviral-BMP2 transduced USCs during bone formation. In fact, previous studies have demonstrated that BMP2 can enhance the ectopic bone formation of bone marrow-derived MSCs [19]. However, the fate of transplanted stem cells *in vivo* differed according to previous studies. Quintavalla *et al*. reported the extensive loss of implanted MSCs on days 7 and 14 in osteochondral defects in goat [20]. Hasegawa *et al*. detected transplanted MSCs four weeks after implantation within the regenerated tissues [21]. In this study, we evaluated the survival of the transplanted cells. The identification of human-derived OCN within the scaffold indicated that some of the transplanted cells survived. We demonstrated that the transplanted cells had survived at six weeks and exerted their osteogenic stimulus on the implantation site to form bone.

Viral vectors currently represent the most efficient approach for gene delivery [22]. A number of viral constructs have been investigated for gene delivery, with the most common including adenovirus, retrovirus, lentivirus and adeno-associated virus [23]. Lentiviral vectors facilitate long-term target gene expression and are attractive with regard to gene therapy [24]. In addition, the immunogenicity associated with Lentiviral vectors is low. How to resolve the safety concerns about Lentiviral vectors is critical for their translation into clinical therapy. Several methods have been proposed to enhance the safety of Lentiviral vectors for gene transduction [25–27]. The results are encouraging and motivate further research to better apply Lentiviral vectors.

## Conclusions

Our study shows that the osteogenic potential of USCs can be significantly enhanced by BMP2 gene transduction. These transfected cells can undergo *in vitro* osteogenic differentiation without osteogenic medium. *In vivo* bone formation assays showed that the Lentiviral-BMP2 transduced USCs provide sufficient stimuli for bone formation. We conclude that the use of BMP2 gene therapy is a useful means of enhancing the osteogenic activity of USCs both *in vitro* and *in vivo*. These results provide a novel strategy for enhancing the osteoproductive ability of USCs.

## Abbreviations

ALP: alkaline phosphatase
ASCs: adipose-derived stem cells
bFGF: basic fibroblast growth factor
BMP2: bone morphogenetic proteins 2
Col I: collagen I
DAPI: 4',6-diamidino-2-phenylindole
(D)MEM: (Dulbecco’s) modified Eagle’s medium
ELISA: enzyme-linked immunosorbent assay
FBS: fetal bovine serum
GFP: green fluorescent protein
H & E: hematoxylin and eosin
hEGF: human epidermal growth factor
MDSCs: muscle-derived stem cells
MOI: multiplicity of infection
OCN: osteocalcin
PBS: phosphate-buffered saline
PDGF: platelet-derived growth factor
PFA: paraformaldehyde
RT-PCR: reverse transcriptase polymerase chain reaction
Runx2: runt-related protein-2
TGF-β: transforming growth factor-β
USCs: human urine-derived stem cells.
